# Immature dendritic cells derived exosomes promotes immune tolerance by regulating T cell differentiation in renal transplantation

**DOI:** 10.18632/aging.102346

**Published:** 2019-10-26

**Authors:** Xin-Lu Pang, Zhi-Gang Wang, Lei Liu, Yong-Hua Feng, Jun-Xiang Wang, Hong-Chang Xie, Xian-Lei Yang, Jin-Feng Li, Gui-Wen Feng

**Affiliations:** 1 Department of Kidney Transplantation, The First Affiliated Hospital of Zhengzhou University, Zhengzhou 450000, China

**Keywords:** miR-682, ROCK2, immature dendritic cells, exosome, T cell differentiation

## Abstract

Objective: To investigate the mechanism of immature dendritic cells-derived exosomes (imDECs) in the regulation of T cell differentiation and immune tolerance in renal allograft model mice.

Results: imDECs significantly improved the percent of survival, relieved inflammatory response, and reduced CD4+T cell infiltration. In addition, imDECs reduced the rejection associated cytokines in allograft mice, and increased the percentage of Foxp3+CD4+T cells in spleen and kidney tissues. imDECs suppressed the IL17+CD4+T cells and promoted the Foxp3+CD4+T cells under Th17 polarization condition. Moreover, miR-682 was found to be highly expressed in imDECs which suppressed the IL17+CD4+T cells and promoted the Foxp3+CD4+T cells. Luciferase reporter assay showed ROCK2 was a target of miR-682, and ROCK mRNA level was negative correlated with miR-682 mRNA level.

Conclusion: miR-682 was highly expressed in imDECs, and imDECs-secreted miR-682 promoted Treg cell differentiation by negatively regulating ROCK2 to promote immune tolerance in renal allograft model mice.

Methods: Renal allograft model mice were established, and imDECs or mature dendritic cells-derived exosomes (mDECs) were injected into model mice. Rejection associated cytokines IFN-γ, IL-2, IL-17 levels in plasma were detected by ELISA. IL-17A, Foxp3, miR-682, ROCK2, p-STAT3, p-STAT5 expressions were measured by qRT-PCR or western blot.

## INTRODUCTION

Kidney transplantation is the most effective treatment for patients with end-stage renal disease (ESRD) [1]. However, it still has challenges such as long-term survival of the recipient and adverse effects of immunosuppressive agents, which induced overall suppression of the immune system and increased infection rate and the incidence of tumor [2, 3]. Regulatory T cells (Tregs) inhibit effector responses via multiple mechanisms, including inhibition of proliferation and activation of CD4+ and CD8+ T cells, suppression B cell responses, and regulation of macrophage and natural killer cell functions [4]. It has been demonstrated that induced Tregs could prevent antibody-mediated renal injury and rejection in a mouse model [5]. Indoleamine 2, 3-dioxgenase (IDO) of mesenchymal stem cells (MSCs) enhanced the function and expression of Treg cells (CD4+Foxp3+T cells) and induced allograft tolerance [6]. Hence, Tregs play a vital role in immune tolerance of kidney transplantation.

Dendritic cells (DCs) are professional antigen-presenting cells that play an important role in triggering T cell-mediated immunity and tolerance [7]. The potential of DCs to trigger immunity or tolerance depends on the maturation status of DC, in which immature DCs (imDCs) induces T-cell tolerance, whereas mature DCs (mDCs) induces T-cell immunity [8]. It has been found that imDC triggered antigen-specific immunotolerance, prolonged renal allograft survival, and increased the content of Tregs in a rat kidney graft model [9]. Moreover, researchers found that exosomes derived from imDCs combined with Tregs induced immune tolerance in a rat liver allograft model and achieved long-term survival [10]. Exosomes derived from imDCs could suppress collagen-induced arthritis and reduce the severity of arthritis, which suggested that imDCs-derived exosomes (imDECs) suppressed inflammatory and autoimmune responses [11]. imDECs could also increase Tregs percentage and Foxp3 mRNA expression in spleen, and prolong the intestinal allograft survival and induce transplant tolerance [12]. Therefore, imDECs could play critical roles in Treg cell differentiation and induction of immune tolerance.

miRNAs exert a post-transcriptional regulatory action on through binding to the 3'-untranslated region (UTR) of the target genes in various biological processes. Studies point out that miRNAs play vital roles in immune responses and Treg cell differentiation [13, 14]. However, the role of exosomes-derived miRNAs in the regulation of immune tolerance in renal allograft survival is still not known. According to the online information of GSE33179, there are remarkable differences in miRNA expressions between imDECs and mDECs, and miR-682 is one of them [15]. Besides, researchers have shown that miR-682 is involved in the NF-κB activation induced inflammatory reaction in intestinal epithelial cells (IECs), and miR-682 overexpression-treated mice has lower TNF-α protein expression in IECs from ischemia-reperfusion mice than sham mice [16], indicating miR-682 is an immune-related miRNA.

Rho-associated kinase 2 (ROCK2) is a member of Rho kinase family that regulates the production of IL-17 and IL-21 and plays an important role in autoimmunity [17]. Recent studies have found that ROCK2 plays an important role in T cell differentiation [18, 19]. ROCK2 promotes Th17 cell differentiation and suppresses Treg differentiation, and abnormal activation of ROCK2 may be involved in the development of autoimmune diseases [17]. KD025 (ROCK2 selective inhibitor) promotes Treg function, shifts Th17/Treg balance, and reduces pro-inflammatory cytokine secretion (IL-17, IL-21, RORγt) in T Cells [18]. Inhibition of ROCK2 remarkably decreases the percentage of IFN-γ+CD4+T cells and IL-17A+CD4+T cells, and increases the percentage of Foxp3+CD4+T cells in inflammatory bowel disease [19]. It has been reported that STAT3 activation promotes Th17 cell differentiation, and STAT5 activation promotes Treg differentiation. ROCK2 inhibitor significantly reduces STAT3 activation and increases STAT5 activation in peripheral blood mononuclear cells [20]. So, ROCK2 may regulate Th17/Treg balance through regulating STAT3 and STAT5 activation. Moreover, bioinformatics database predicted that ROCK2 was a candidate target of miR-682. Therefore, we speculated that imDECs-secreted miR-685 decreased ROCK2 expression in CD4+T cells, thereby inducing Treg cell differentiation and immune tolerance in kidney transplantation.

## RESULTS

### imDEC relieved acute rejection after kidney transplantation

**Figure 1:**
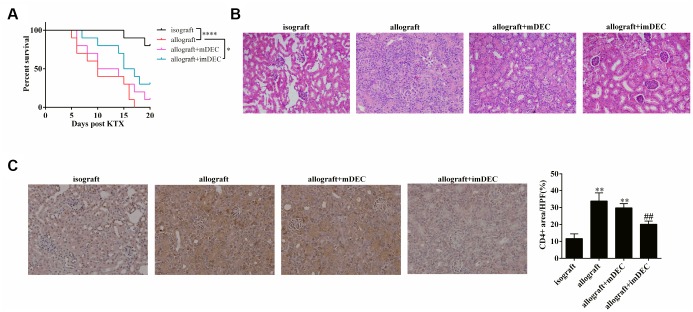
**imDEC relieved acute rejection after kidney transplantation.** Mice were divided into four groups: isograft group (n=10), allograft group (n=10), allograft+mDEC group (n=10), and allograft+imDEC group (n=10). (**A**) The percent of survival in isograft group, allograft group, allograft+mDEC group, and allograft+imDEC group. ****P<0.0001 vs isograft group; *P<0.05 vs allograft group. (**B**) H&E staining of kidney tissue (n=7) showed that the inflammatory response was relieved in allograft+imDEC group compared with allograft group. (400×magnification). (**C**) CD4 staining of kidney tissue (n=7) showed that CD4+T cell infiltration was reduced in allograft+imDEC group. Image J was used to analyze the images and calculate the CD4+ area. (400×magnification). **P<0.001 vs isograft group; ##P<0.001 vs allograft+mDEC group.

As shown in Figure 1A, the percent of survival in isograft mice was significantly higher than the allograft mice. The injection of imDECs significantly improved the percent of survival, whereas the injection of mDEC didn’t significantly change the percent of survival (Figure 1A). The median survival time of allograft group, allograft+mDEC group, and allograft+imDEC group was 10 d, 12 d, and 16 d (Figure 1A). H&E staining of kidney tissue showed that the inflammatory response was relieved in allograft+imDEC group compared with allograft group (Figure 1B). In addition, CD4 staining of kidney tissue showed that CD4+T cell infiltration was significantly reduced in allograft+imDEC group (Figure 1C). The percentage of CD4+ area was significantly decreased in allograft+imDEC group than allograft or allograft+mDEC group (Figure 1C). These findings indicated that the injection of imDEC relieved acute rejection after kidney transplantation.

### imDEC reduced the production of cytokines

**Figure 2:**
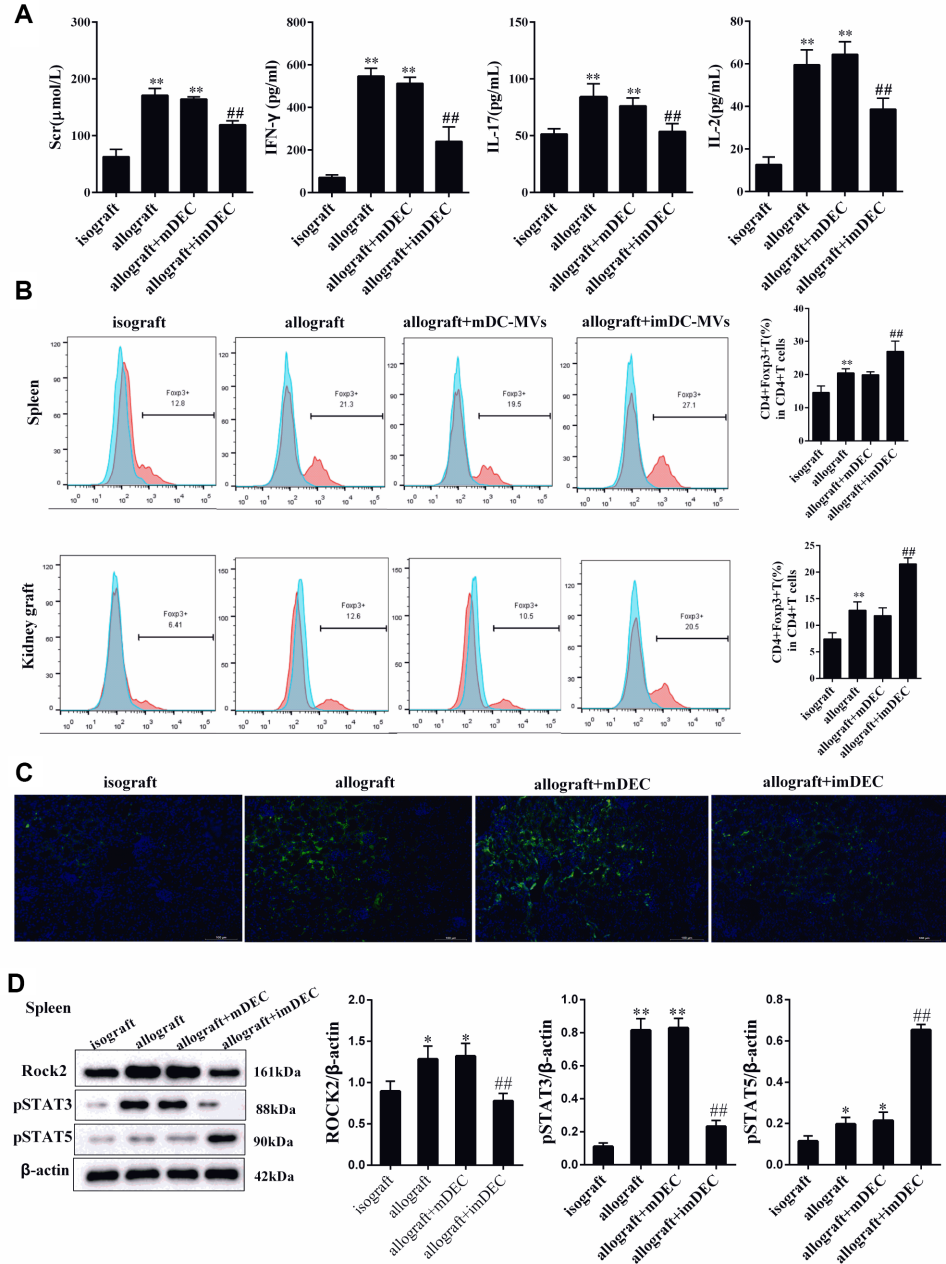
**imDEC reduced the production of cytokines.** (**A**) Renal function indicator Scr, and rejection associated cytokines IFN-γ, IL-2, IL-17 levels in plasma were detected in isograft group, allograft group, allograft+mDEC group, and allograft+imDEC group. (**B**) The percentage of Foxp3+CD4+T cells in spleen and kidney tissues was detected in isograft group, allograft group, allograft+mDEC group, and allograft+imDEC group. (**C**) Immunofluorescence images show the expression of ROCK2 in isograft group, allograft group, allograft+mDEC group, and allograft+imDEC group. (**D**) The expression of ROCK2, p-STAT3 and p-STAT5 were measured in CD4+T cells from spleen of isograft group, allograft group, allograft+mDEC group, and allograft+imDEC group. **P<0.01 vs isograft group; ##P<0.01 vs allograft+mDEC group.

Cytokines are important mediators in the induction of immune response in kidney transplantation. Herein, the levels of rejection associated cytokines in the grafts of recipients were measured. We found renal function indicator serum Scr, and rejection associated cytokines IFN-γ, IL-2, IL-17 levels in plasma were significantly up-regulated in allograft mice than that of isograft mice (Figure 2A). And the injection of imDEC significantly reduced the rejection associated cytokines in allograft mice (Figure 2A). Compared with allograft group, the percentage of Foxp3+CD4+T cells in spleen and kidney tissues was significantly higher in allograft+imDEC group, whereas there was no significant change in the percentage of Foxp3+CD4+T cells between allograft+mDEC group and allograft group (Figure 2B). According to the immunofluorescence images, the expression of ROCK2 in allograft group was higher than isograft group, and the expression of ROCK2 in allograft+imDEC group was reduced than allograft group (Figure 2C). Next, we detected the ROCK2, STAT3, STAT5 expressions in the spleen. The expression of ROCK2 and p-STAT3 were significantly up-regulated in CD4+T cells from spleen of allograft mice, whereas p-STAT5 expression was significantly down-regulated in CD4+T cells from spleen of allograft mice (Figure 2D). The injection of imDEC could decrease the expression of ROCK2 and p-STAT3, and increase the expression of p-STAT5 (Figure 2D).

### imDEC suppressed the IL17+CD4+T cells and promoted the Foxp3+CD4+T cells

**Figure 3:**
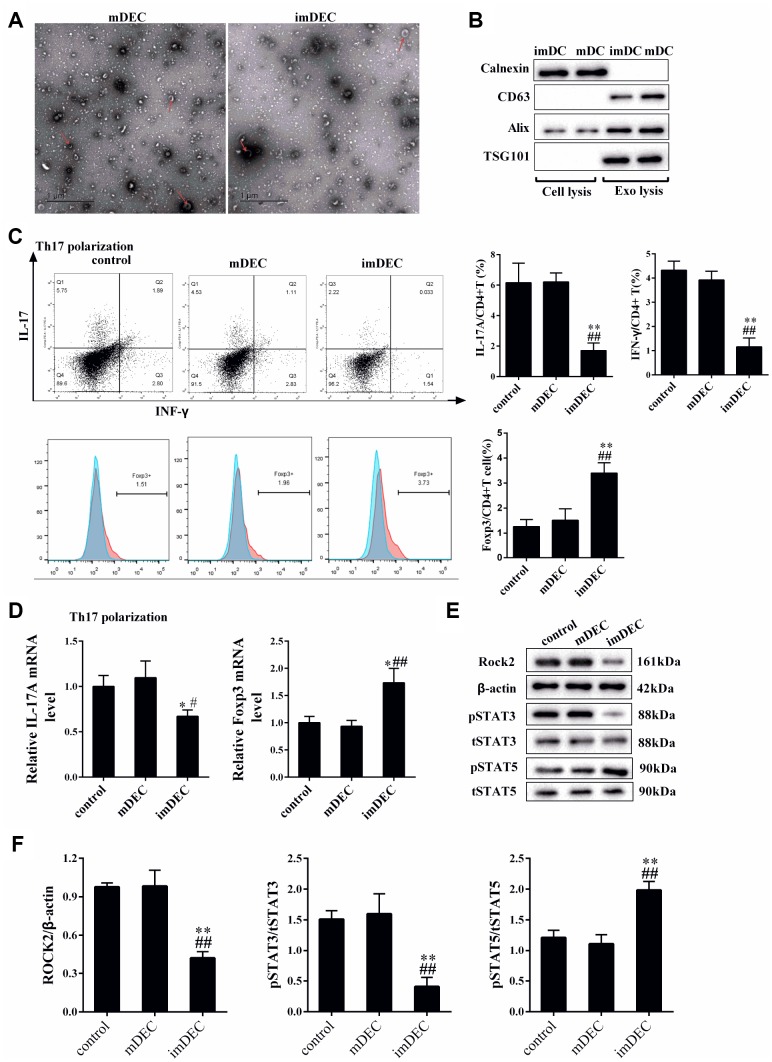
**imDEC suppressed the IL17+CD4+T cells and promoted the Foxp3+CD4+T cells.** (**A**) imDECs and mDECs from imDCs and mDCs were determined by transmission electron microscope (TEM). (**B**) Western blot showed the Calnexin, exosome markers CD63, Alix and TSG10 expressions in cell lysis and exo lysis of imDCs and mDCs. (**C**) Primary CD4+T cells were collected from the spleen. Under Th17 polarization condition, the percentage of IL17+CD4+T cells and IFN-γ+CD4+T cells were detected in control, mDEC and imDEC groups. (**D**) The mRNA transcription of Foxp3 and mRNA transcription of IL-17A were detected in control, mDEC and imDEC groups under Th17 polarization condition for 72 h. (**E**–**F**) The expressions of p-STAT5, p-STAT3 and ROCK2 were detected in control, mDEC and imDEC groups. *P<0.05 vs control; **P<0.01 vs control; #P<0.05 vs mDEC; ##P<0.01 vs mDEC.

As shown in Figure 3A and 3B, imDECs and mDECs from imDCs and mDCs were determined by transmission electron microscope or western blot. Calnexin was detected in cell lysis of imDCs and mDCs, whereas it was not detected in exo lysis of imDCs and mDCs (Figure 3B). In addition, exosome markers CD63, Alix and TSG101 were found in in exo lysis of imDCs and mDCs, whereas they were not found in cell lysis of imDCs and mDCs (Figure 3B). Next, we determined the effect of imDEC on the differentiation of IL17+CD4+T cells and Foxp3+CD4+T cells, and primary CD4+T cells were collected from the spleen. Under Th17 polarization condition, imDEC suppressed the percentage of IL17+CD4+T cells and IFN-γ+CD4+T cells, and promoted the percentage of Foxp3+CD4+T cells (Figure 3C). Besides, imDEC promoted the mRNA transcription of Foxp3 and inhibited the mRNA transcription of IL-17A under Th17 polarization condition for 72 h (Figure 3D). imDEC also promoted the expression of p-STAT5, and inhibited the expression of p-STAT3 and ROCK2 (Figure 3E and 3F).

### miR-682 was highly expressed in imDEC which suppressed the IL17+CD4+T cells and promoted the Foxp3+CD4+T cells

**Figure 4:**
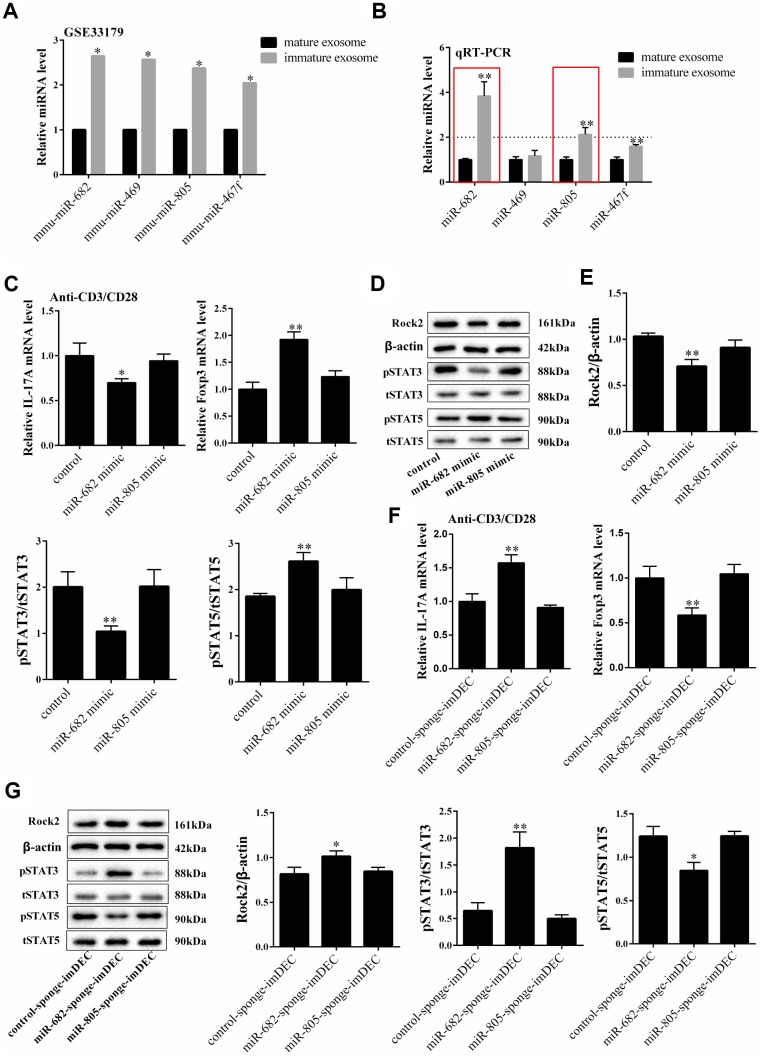
**miR-682 was highly expressed in imDEC which suppressed the IL17+CD4+T cells and promoted the Foxp3+CD4+T cells.** (**A**) According to the information of GSE33179, the expressions of miR-682, miR-649, miR-805 and miR-467f in imDEC and mDEC. (**B**) qRT-PCR detected the expressions of miR-682, miR-649, miR-805 and miR-467f in imDEC and mDEC. *P<0.05 vs mature exosome; **P<0.01 vs mature exosome. (**C**) Under anti-CD3/CD28 activated condition, mRNA level of IL-17A and mRNA level of Foxp3 were detected in control, miR-682 mimic and miR-805 mimic groups. ROCK2, p-STAT3 and p-STAT5 expressions in control, miR-682 mimic and miR-805 mimic groups. *P<0.05 vs control; **P<0.01 vs control. (**D-E**) The expression of STAT5, STAT3 and ROCK2 were measured in control, miR-682 mimic and miR-805 mimic groups. (<bold>F</bold>) Under anti-CD3/CD28 activated condition, mRNA level of IL-17A and mRNA level of Foxp3 were detected in control-sponge-imDEC, miR-682-sponge-imDEC and miR-805-sponge-imDEC groups. (<bold>G</bold>) The expression of STAT5, STAT3 and ROCK2 were measured in control-sponge-imDEC, miR-682-sponge-imDEC and miR-805-sponge-imDEC groups. The expression of p-STAT5, p-STAT3 and ROCK2 were measured in control-sponge-imDEC, miR-682-sponge-imDEC and miR-805-sponge-imDEC groups. **P<0.01 vs control-sponge-imDEC.

According to the information of GSE33179, the expressions of miR-682, miR-649, miR-805 and miR-467f were higher in imDEC than mDEC (Figure 4A). qRT-PCR showed that miR-682 and miR-805 were highly expressed in imDEC than mDEC (Figure 4B). Under anti-CD3/CD28 activated condition, miR-682 overexpression significantly decreased mRNA level of IL-17A and increased mRNA level of Foxp3, which had similar effect as imDEC (Figure 4C). However, miR-805 overexpression did not change the mRNA level of IL-17A and Foxp3. Besides, miR-682 mimic down-regulated ROCK2 and p-STAT3 expressions, and up-regulated p-STAT5 expression (Figure 4D and 4E). Under anti-CD3/CD28 activated condition, miR-682-sponge-imDEC promoted mRNA level of IL-17A and inhibited mRNA level of Foxp3 (Figure 4F). In addition, miR- 682-sponge-imDEC significantly promoted ROCK2, p-STAT3 expression, and decreased p-STAT5 expression (Figure 4G). However, miR-805-sponge-imDEC didn’t significantly change the expressions of ROCK2, p-STAT3 and p-STAT5. These findings indicated that miR-682 might be a mediator of imDEC regulating T cell differentiation.

### ROCK2 was a target of miR-682

**Figure 5:**
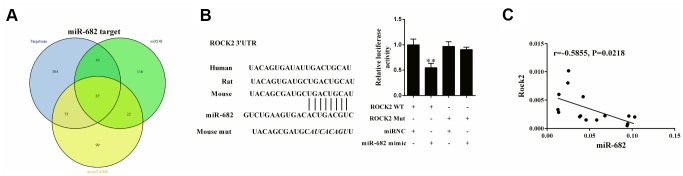
**ROCK2 was a target of miR-682.** (**A**) Thirty-seven common candidates for the targeted mRNAs of miR-682 were found in the Targetscan, miRDB, and microT-CDS database using Venn diagram analysis. VennPlex version 1.0.0.2 software was used for Venn diagram analysis. Proportional circular sets will be drawn if three data sets are uploaded. (**B**) The binding sites between miR-682 and ROCK2 3’UTR. Luciferase reporter assay was conducted to show the regulation of miR-682 in ROCK2. **P<0.01 vs ROCK2 WT+miRNA. (**C**) T cells were collected from the spleen in allograft group, allograft+imDEC group and allograft+mDEC group to detect the correlation of miR-682 and ROCK2 mRNA.

The targeted mRNAs of miR-682 were analyzed by three online bioinformatics database (Targetscan, miRDB, and microT-CDS), and there were 37 common candidates in the three database using Venn diagram analysis for evaluation based on R language (Figure 5A). ROCK was one of these mRNAs, and the binding sites between miR-682 and ROCK2 3′UTR were shown in Figure 5B. Luciferase reporter assay showed that miR-682 mimic significantly suppressed luciferase activity of ROCK2 WT, indicating that miR-682 targetedly regulated ROCK2 (Figure 5B). T cells were collected from the spleen in allograft group, allograft+imDEC group and allograft+mDEC group to detect the correlation of miR-682 and ROCK2 mRNA. We found ROCK was negative correlated with miR-682, which further proved the negative role of miR-682 on ROCK2 (Figure 5C).

### miR-682 regulated T cell differentiation via ROCK2 under Th17 polarization condition

**Figure 6:**
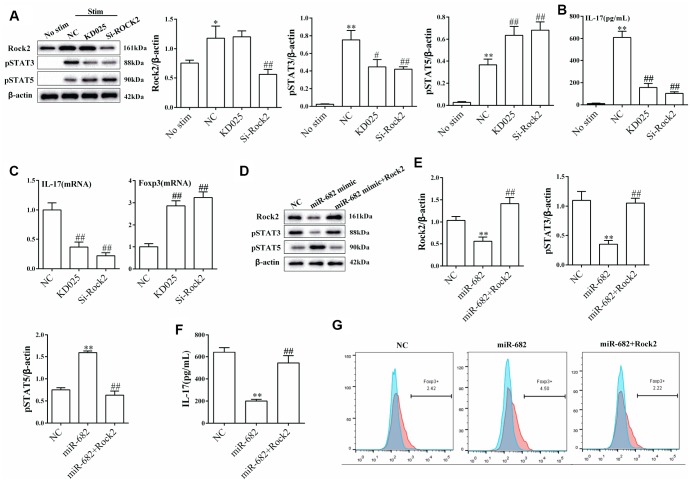
**miR-682 regulated T cell differentiation via ROCK2 under Th17 polarization condition.** T cells were divided into no stimulation group, NC group (under Th17 polarization stimulation), ROCK2 selective inhibitor KD025 group (under Th17 polarization stimulation) and si-ROCK2 group (under Th17 polarization stimulation). (**A**) The expressions of ROCK2, p-STAT3 and p-STAT5 were measured by western blot in the four groups. (**B**) IL-17 secretion was detected by ELISA in the four groups. **P<0.01 vs no stim; ##P<0.01 vs NC. (**C**) IL-17 mRNA level and Foxp3 mRNA level were detected by qRT-PCR. ##P<0.01 vs NC. T cells were transfected with NC, miR-682 mimic or miR-682 mimic+ROCk2. (**D**–**E**) The expression of ROCK2, p-STAT3 and p-STAT5 were detected by western blot. (**F**) IL-17 secretion was detected by ELISA. **P<0.01 vs NC; ##P<0.01 vs miR-682. (**G**) The percentage of Foxp3+CD4+T cells was detected by flow cytometry.

Under Th17 polarization stimulation, si-ROCK significantly suppressed the expression of ROCK2. KD025 (ROCK2 selective inhibitor) and small interference RNA targeting ROCK2 (si-ROCK2) significantly decreased the expression of p-STAT3 and increased the expression of p-STAT5 (Figure 6A). Moreover, KD025 and si-ROCK2 significantly decreased IL-17 secretion and mRNA level, and increased Foxp3 mRNA level (Figure 6B and 6C). miR-682 mimic down-regulated the expression of ROCK2 and p-STAT3, inhibited IL-17 secretion, up-regulated the expression of p-STAT5, and promoted the percentage of Foxp3+CD4+T cells (Figure 6D–6G). And overexpression of ROCK2 could reverse the effect of miR-682 on the regulation of T cell differentiation (Figure 6D–6G).

### imDEC regulated acute rejection after kidney transplantation through miR-682

**Figure 7:**
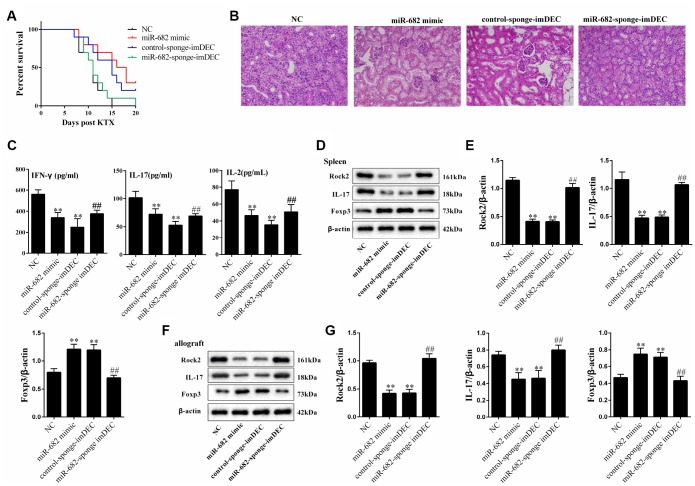
**imDEC regulated acute rejection after kidney transplantation through miR-682.** 10 μg control-sponge-imDEC, miR-682-sponge-imDEC or miR-682 mimic was injected into the allograft model mice via the tail vein 24 hours before and after transplantation (ten mice in each group). Allograft mice were used as NC (n=10). (**A**) The survival rate in control-sponge-imDEC group, miR-682-sponge-imDEC group, miR-682 mimic group and NC group. (**B**) H&E staining showed that the remission effect of miR-682-sponge-imDEC on renal inflammation, indicating that imDEC could regulate acute rejection after kidney transplantation through miR-682. (**C**) Serum IL-2, IL-17 and IFN-γ levels were detected in control-sponge-imDEC group, miR-682-sponge-imDEC group, miR-682 mimic group and NC group.**P<0.01 vs NC; ##P<0.01 vs control-sponge-imDEC. (**D**–**G**) The proteins of T cells were extracted from the spleen and allograft kidney tissues, and the ROCK2, IL-17 and Foxp3 expressions were detected in control-sponge-imDEC group, miR-682-sponge-imDEC group, miR-682 mimic group and NC group.

To demonstrate the effect of imDEC-derived miR-682 on survival rate, 10 μg control-sponge-imDEC, miR-682-sponge-imDEC or miR-682 mimic was injected into the allograft model mice via the tail vein 24 hours before and after transplantation (ten mice in each group). We found that the survival rate in miR-682-sponge-imDEC group was lower than control-sponge-imDEC group, and the survival rate in miR-682 mimic group and control-sponge-imDEC group was higher than NC group (Figure 7A). H&E and CD4 staining showed that the remission effect of miR-682-sponge- imDEC on renal inflammation indicating that imDEC could regulate acute rejection after kidney transplantation through miR-682 (Figure 7B). In addition, serum IL-2, IL-17 and IFN-γ levels were significantly reduced in miR-682 mimic group and control-sponge-imDEC group, whereas miR-682-sponge-imDEC increased serum IL-2, IL-17 and IFN-γ levels compared with control-sponge-imDEC (Figure 7C). We further extracted the proteins of T cells from the spleen and allograft kidney tissues, and detected the ROCK2, IL-17 and Foxp3 expressions. The effect of miR-682-sponge-imDEC on inhibiting IL-17 and promoting Foxp3 was reduced (Figure 7D–7G).

## DISCUSSION

Studies have reported that DC-derived exosome could play important roles in immune regulation, such as binding Toll-like-receptor ligands to innate-immunity effector cells, or inducing the specific anti-tumor immune response [26, 27]. imDC is a status of DC that induces T-cell tolerance, which can prolong the survival of renal allograft and increase Treg numbers in a rat kidney graft model [9]. Immune tolerance and long-term survival were also achieved in a rat liver allograft model after combined treatment of imDECs and Tregs [10]. In addition, imDECs increased the percentage of Tregs and Foxp3 mRNA expression in the spleen and induced transplant tolerance [12]. In this study, we purified exosomes from mDC and imDC and injected them into renal allograft model mice. We determined imDECs remarkably increased survival rate, decreased rejection associated cytokines IFN-γ, IL-2, IL-17 levels, promoted the percentage of Foxp3+CD4+T cells in allograft mice, which indicated imDECs were involved in the induction of immune tolerance in renal allograft model mice. Whereas mDECs could not remarkably change the survival rate, rejection associated cytokines levels or the percentage of Treg cells, indicating mDECs could not induce immune tolerance in renal allograft model mice. To the best of our knowledge, no other studies have demonstrated the role of imDECs in kidney transplantation of renal allograft model or compared the effect of imDEC and mDEC on kidney transplantation. The findings enrich the literature and provide directions for the treatment of immune rejection after kidney transplantation.

miRNAs are emerging as vital modulators of gene expression in the immune response which control the differentiation and function of Treg cells [28, 29]. Evidence also showed that DECs could secrete miRNAs (miR-155 and miR-146a) to regulate inflammatory response to LPS [30]. So the DECs secreted miRNAs might be involved in the regulation of Tregs differentiation and function. In this study, we detected the expressions of miR-682, miR-649, miR-805 and miR-467f in imDEC and mDEC, and found that miR-682 and miR-805 showed abnormal expressions in imDEC and mDEC. After miR-682 and miR-805 overexpression or blockade of exosomal miR-682 and miR-805, we further found only the abnormal expression of miR-682 changed the IL-17A and Foxp3 mRNA levels, which indicated that only miR-682 might be involved in the regulation of Tregs differentiation. So, we focused on the role of miR-682 in kidney transplantation, and found miR-682 was highly expressed in imDECs. exosomal miR-682 overexpression decreased IL-17A mRNA level and increased Foxp3 mRNA level, and the blockade of exosomal miR-682 achieved the opposite effect. Herein, we first proved that imDECs-secreted miR-682 could promote Tregs differentiation to induce immune tolerance. In addition, we further determined the underlying mechanism of exosomal miR-682 in the regulation of Tregs differentiation after kidney transplantation.

Recently, several studies have proved that ROCK2 inhibition promoted Tregs differentiation [18, 29]. In this study, we demonstrated that silencing ROCK2 reduced IL-17 level and increased Foxp3 level, which was consistent with previous reports [18, 29]. Luciferase reporter assay showed miR-682 could target to 3’ UTR of ROCK2, and ROCK2 mRNA level was negatively correlated with miR-682 mRNA level. Moreover, we found exosomal miR-682 suppressed IL-17 secretion and p-STAT3, and up-regulated p-STAT5 through regulating ROCK2. All these findings indicated imDECs-secreted miR-682 induced STAT3 and STAT5-dependent Treg cell differentiation by inhibiting ROCK2 in CD4+ T cells. *In vitro* experiments showed exosomal miR-682 blockade reduced the remission effect of imDEC on renal inflammation and promotion effect of Treg differentiation, indicating that imDEC regulated acute rejection after kidney transplantation through secreting miR-682.

In conclusion, this study demonstrated that miR-682 was highly expressed in imDECs, and imDECs-secreted miR-682 promoted Treg cell differentiation by negatively regulating ROCK2 in CD4+ T cells. *In vitro* experiments proved that the blockade of exosomal miR-682 reduced the survival rate, increased the secretion of rejection associated cytokines and IL-17 expression, and decreased Foxp3 expression, which indicated that imDECs-secreted miR-682 promoted immune tolerance in renal allograft model mice.

## MATERIALS AND METHODS

### Establishment of renal isograft and allograft model mice

All animal experiments were approved by Ethic Committee of The First Affiliated Hospital of Zhengzhou University. For the establishment of mice allograft kidney transplantation, male C57BL/6 mice were used as kidney donors and female BALB/c mice were used as recipients under isoflurane inhalation anesthesia (Sinopharm Chemical Reagent, China). For isogenic kidney transplantation, BALB/c mice were used as kidney donors and recipients under isoflurane inhalation anesthesia and subcutaneously injected the butorphanol (1mg/kg). In donor mice, the left kidney, the aorta and inferior vena cava and the ureter were removed. Then, in recipient mice, the left kidney was removed, and the allograft was placed into the left lower abdomen. The vessels of the allograft were anastomosed to the aorta and inferior vena cava of recipient, and the ureter was implanted into the bladder. The time for cold ischemia and warm ischemia time was 60 and 20 min, respectively. Mice were divided into isograft group (n=10), allograft group (n=10), allograft+mDEC group (n=10) and allograft+imDEC group (n=10). In isograft group, BALB/c mice were used as kidney donors and recipients. In allograft group, C57BL6 mice were used as kidney donors and BALB/c mice were used as recipients. Contralateral nephrectomy was performed at the time of kidney transplantation, so the mice survived only on transplanted kidneys. In allograft+mDEC group, 10 μg mDECs (exosome from mature DC) were injected into the recipient mice via the tail vein 24 hours before and after transplantation. In allograft+imDEC group, 10 μg imDECs (exosome from immature DC) were injected into the recipient mice via the tail vein 24 hours before and after transplantation. All the recipient mice were single-pass injected with Ampicillin. Mice (seven mice in each group) were sacrificed six days after transplantation, and kidneys were collected for hematoxylin and eosin (H&E) and CD4 staining. Blood was also collected from inferior vena cava on the 6^th^ day after transplantation for the measurement of renal function.

To observe the effect of exosome-derived miR-682 on acute rejection after kidney transplantation, control-sponge-imDEC, miR-682-sponge-imDEC, or miR-682 mimic (10 μg) was injected into the allograft model mice via the tail vein 24 hours before and after transplantation (ten mice in each group). Allograft mice (n=10) were used as negative control (NC). Mice (five mice in each group) were sacrificed six days after transplantation, and kidneys and spleens were collected for H&E and CD4 staining. Blood was also collected from inferior vena cava on the 6^th^ day after transplantation for the measurement of renal function.

### H&E and CD4 staining

Kidney tissues were fixed in 10% neutral buffered formalin for 48 h, dehydrated in ethanol, embedded in paraffin, and cut into 5 μm slices. Slices were prepared and stained with H&E, and observed under a light microscope (Olympus, Japan). CD4 (1:200; Abcam, USA) was stained according to the manufacturer's instructions.

### Renal function analysis

Serum creatinine (SCr) level was measured by enzymatic-colorimetric method using an automatic biochemistry analyzer (BS-480; Mindray, China). Plasma IFN-γ, IL-2 and IL-17 levels were detected by ELISA assay (Signalway antibody, USA) according to the manufacturer’s instructions.

### Generation and culture of immature DCs

Immature DCs were generated from bone marrow flushed from the femurs and tibias of recipient mice as previously reported with 20ng/ml recombinant mouse IL-4 (eBioscience, USA) and 30ng/ml recombinant mouse GM-CSF (eBioscience, USA) [21]. Cells were cultured from day 8 to 10 without cytokines. Then, non-adherent and adherent cells were harvested at day 10. After removal of adherent cells, cells were identified as imDCs, and then cultured from day 10 to 12 with 200 ng/mL LPS (Sigma, USA) to obtain mDCs.

### Isolation of exosomes

mDCs and imDCs were cultured for 3 days, and 1×10^6^ DCs were collected in the DMEM-Low Glucose medium (Gibco, USA). The medium containing DCs was centrifuged at 300×g, 1200×g and 10,000×g for 10 min, 20 min, and 30 min to remove large fragments. Then, the exosome pellet was washed in saline, passed through a 0.22 μm filter, centrifuged at 100,000×g for 60 min. After determined with CD63 and CD81, the pellet was resuspended in 2 ml PBS. About 15 μg exosomes were harvested from 1×10^7^ mDCs or imDCs. The amount of exosomes (imDECs, exosome from immature DC; mDECs, exosome from mature DC) was evaluated based on the amount of protein using Pierce™ BCA Protein Assay Kit (Thermo Scientific, USA).

### Electron microscopy

Exosomes were diluted ten times, loaded on a Formvar/carbon coated grid, negatively stained with 10 μl of neutral 1% aqueous phosphotungstic acid, and viewed using an electron microscope (CM120; Philips, Netherlands).

### Isolation of CD4+T cells

Spleen was dissociated through a 40 μm nylon mesh and then cultivated in DMEM medium containing 10% fetal bovine serum (FBS; Gibco, USA). MagniSort™ Mouse CD4 T cell Enrichment Kit (Invitrogen, USA) was used to isolate CD4+T cells according to the manufacturer’s instructions.

CD4+T cells were isolated from kidney tissues according to previous reports [22, 23]. Kidney cortex was minced and digested with collagenase (Gibco, USA) and deoxyribonuclease (Sigma, USA), and then passed through a 40 μm nylon mesh. Cells were obtained by centrifugation, resuspended in Percoll (40%; Sigma, USA) in RPMI-1640 medium (Gibco, USA), and overlayed on Percoll (66.7%; Sigma, USA). Gradient separation was centrifugated at 800×g for 20 min at 25°C. Cell purity was >95%.

### Flow cytometer

CD4+T cells isolated from spleen and kidney were resuspended at 3×10^6^ cells/ml. Cells were detected by BD FACSCanto II flow cytometry (BD, USA) and analyzed using CELLQuest software. eBioscience™ Mouse Regulatory T Cell Staining Kit (Invitrogen, USA) was used for the observation of CD4+Foxp3+ T cells. Anti-IL-17A was purchased from eBioscience (ThermoFisher Scientific, USA) for the identification of IL-17A+CD4+T cells.

### Western blot

Kidney or spleen tissues and CD4+T cells were lysed in Radio Immunoprecipitation Assay (RIPA) buffer (Thermo Scientific, USA). Protein was separated by SDS-polyacrylamide gel electrophoresis (PAGE), then transferred to polyvinylidene fluoride (PVDF) membrane (Invitrogen, USA). Blots were incubated with primary antibodies against ROCK2 (1:500; Abcam, USA), p-STAT3 (1:2000; Abcam, USA), p-STAT5 (1:500; Abcam, USA), Calnexin (1:2000; Abcam, USA), CD63 (1:500; Abcam, USA), Alix (1:1000; Abcam, USA), TSG101 (1:2000; Abcam, USA), β-actin (Sigma, USA) and horseradish peroxidase-conjugated secondary antibody (Abcam, USA). β-actin was used as an internal control. Blots were visualized by Molecular Imager® Gel Doc™ XR System (Bio-Rad, USA).

### Quantitative real-time RCR (qRT-PCR)

Total RNAs were isolated from CD4+T cells using Trizol (Invitrogen, USA), and inversely transcribed into cDNA using the High-Capacity cDNA Reverse Transcription Kit with RNase Inhibitor (Invitrogen, USA). qRT-PCR was conducted to measure IL-17A, Foxp3, ROCK2 expression using PowerUp™ SYBR™ Green Master Mix (Invitrogen, USA). miR-682, miR-649, miR-805 and miR-467f expressions were detected using Mir-X miRNA First-Strand Synthesis kit (TAKARA, Japan). The relative expression of IL-17A, Foxp3, ROCK2, miR-682, miR-649, miR-805 and miR-467f were expressed as a function of threshold cycle (Ct) and analyzed by 2^-ΔΔCt^ method. GAPDH or U6 was used as internal reference. The primer sequences were as follows: IL-17A: (F): 5′-TTTAACTC CCTTGGCGCAAAA-3′, ®: 5(R)-CTTTCCCTCCGCATT GACAC-3′; Foxp3: (F): 5′- GGCCCTTCTCCAGGAC AGA-3′, ®: 5(R)- GCTGATCATGGCTGGGTTGT-3′; ROCK2: (F): 5′-AAAACTGTGATCCCAAGGGAAG-3′, ®: 5(R)-CACATGAACTGAGCAAAGCCC-3′; GAPDH: (F): 5(R)- TGGATTTGGACGCATTGGTC-3′, ®: 5′- TTTGCACTGGTACGTGTTGAT-3′; mmu-miR-467f: cgcggATATACACACACACACCTACA; mmu-miR-805: acggcgGAATTGATCAGGACATAG; mmu-miR-469: cgcgCCTCTTTCATTGATCTTGG; mmu-miR-682: cgCTGCAGTCACAGTGAAGTCTG.

### Transfection with lentiviral vector

ViraPower™ II Lentiviral Gateway™ Expression System (Invitrogen, USA) was used to construct Lentiviral expression vectors as previously reported [24]. CD4+T cells were transfected with lentivirus-mediated miR-682 mimic, miR-805 mimic lentiviral vectors with 8 μg/mL polybrene (Sigma, USA). Scramble sequence was set as negative control. Cells were cultured in medium containing 1μg/mL puromycin (Sinopharm Chemical Reagent Co., China) for selection 24 h later.

### The blockade of exosomal miR-682 and miR-805

The sequences of the sponges were as follows: Sponge for miR-682: 5′-CAGACUUCACUGUGACUGCAG-3′; Sponge for miR-805: 5′-CUAUGUCCUGAUCAA UUC-3’; negative control: 5′-CGAACUUCACUGTGA CUGCAG-3′. The constructs (vector + linkers sponges) were ligated in one reaction using a vector:linker ratio of 1:3 and a vector:sponge ratio of 1:1000. The ligation reaction was performed using T4 DNA ligase (New England BioLabs, Inc.). The miR-682-sponge-imDEC (blockade of exosomal miR-682) was generated from miR-682-sponge-imDC, in which miR-682 was knockdown by transfering a lentivirus-packaged sponge RNA targeting miR-682 (lenti-miR-682-sponge). Control-sponge-imDEC was generated from imDC transfected with a lentivirus-packaged sponge RNA targeting a control gene (lenti-control-sponge), which was used as a control. The preparation of miR-805-sponge-imDEC was the same as miR-682-sponge-imDEC.

### Luciferase reporter assay

Luciferase report gene vectors (pRL-TK, Promega) containing 400ng ROCK2 Wild Type (WT) or 400 ng ROCK2 mutant (Mut) were transfected into 293T cells. miR-682 mimic or negative control (miRNC) were co-transfected with reporter plasmids for 48 h. 293T cells were collected to measure luciferase activity by Dual-Luciferase Reporter Assay System (Promega).

### Th17 polarization stimulation

CD4+T cells (2×10^6^/well) were cultured in a 24-well plate containing 30 ng/ml IL-6, 10 ng/ml IL-1β, 100 ng/ml IL-23, 20 μg/ml anti-INFγ, 10 μg/ml anti-IL-4, 10 μg/ml anti-IL-2, and 0.1 ng/ml recombinant mouse TGF-β1 (Abcam, USA) for 3 days [25].

### Statistical analysis

The data corresponded to mean±standard errors (SE), and were analyzed by SPSS software (version 18.0). Results were analyzed using *t* test or one-way analysis of variance (ANOVA), with p<0.05 considered statistically significant.
